# Massive Bilateral Hydroureteronephrosis and End-Stage Renal Disease Ensuing From Chronic Schistosomiasis: A Case Report

**DOI:** 10.1177/2324709620910912

**Published:** 2020-03-05

**Authors:** Pedro Pallangyo, Smita Bhalia, Nontobeko Nokulunga Simelane, Frederick Lyimo, Happiness Judical Swai, Zabella Seifu Mkojera, Naairah Rashid Hemed, Nsajigwa Misidai, Jalack Millinga, Mohamed Janabi

**Affiliations:** 1 Jakaya Kikwete Cardiac Institute, Dar es Salaam, Tanzania; 2 Mbabane Government Hospital, Mbabane, Swaziland; 3 Muhimbili National Hospital, Dar es Salaam, Tanzania

**Keywords:** hydroureteronephrosis, hydronephrosis, hydroureter, schistosomiasis, bilharzia, snail fever, neglected diseases, tropical diseases, parasitic diseases

## Abstract

Globally, schistosomal infections affect over 200 million people resulting in the loss of 70 million disability-adjusted life years. In the sub-Saharan Africa region, where over 85% of the global schistosomal infections are found, it is estimated that about 120 million people become symptomatic, over 20 million have severe disease, and nearly 200 000 die every year. Renal impairment is a severe consequence of schistosomiasis occurring in about 6% of all infected individuals and in 15% of those with the hepatosplenic form. We present a case of massive bilateral hydroureteronephrosis and end-stage renal disease resulting from chronic schistosomiasis in a 38-year-old male of African origin. A 38-year-old male rice farmer of African origin presented with a history of elevated blood pressure, abdominal swelling, and reduced urinary output for about 10 months. Abdominal examination revealed an intraabdominal mass measuring 30 cm × 17 cm extending from the right hypochrondrium region downward to right inguinal outward to umbilicus crossing the midline. He had an estimated glomerular filtration rate of 3.9 mL/min, hemoglobin of 6.78 g/dL, and had multiple electrolyte abnormalities. A computed tomography intravenous urogram scan of the abdomen revealed hepatomegaly (18 cm), bilateral renal enlargement with hydroureteronephrosis, and multiple calcifications on the urinary bladder. A rectal biopsy isolated *haematobium* eggs and confirmed the diagnosis. Urinary schistosomiasis can have distressing effects on the urinary system in particular and survival prospects in general. In view of this, extensive evaluation of the genitourinary system is pivotal for timely diagnosis and prompt management particularly in residents of schistosoma-endemic communities presenting with obstructive uropathy.

## Introduction

Schistosomiasis is a devastating water-borne parasitic infection that affects about 3.5% of the global population.^1^ This neglected chronic debilitating illness poses a substantial socioeconomic burden in the tropical world and is ranked second to malaria among parasitic infections of public health importance.^2^ Also known as bilharzia, this infectious disease of poverty is notorious for causing destructive granulomatous inflammation with intense fibrocollagenous tissue deposition in various body organs.^3^ The schistosoma species that causes disease in humans include *haematobium, intercalatum, japonicum, mansoni*, and *mekongi*.^4^ In the sub-Saharan Africa region, where over 85% of the global schistosomal infections are found, it is estimated that about 120 million people become symptomatic, over 20 million have severe disease, and nearly 200 000 die every year.^4-6^

Similar to many other helminthic diseases, schistosomiasis exhibit a wide spectrum of renal manifestations, including chronic granulomatous disease, chronic pyelonephritis, obstructive renal failure, and schistosomal glomerulopathy.^3,7^ Renal impairment is a severe consequence of schistosomiasis occurring in about 6% of all infected individuals and in 15% of those with the hepatosplenic form.^4,8^ It is estimated that renal failure ensuing from schistosomiasis claims about 150 000 lives worldwide every year.^2^ Thus far, antiparasitic regimens used in the management of schistosomiasis have shown no effect whatsoever on the progressive evolution of renal injury to end-stage renal disease (ESRD).^9,10^ We present a case of massive bilateral hydroureteronephrosis and ESRD resulting from chronic schistosomiasis in a 38-year-old male of African origin.

## Case Description

A 38-year-old male rice farmer of African origin was referred to us from an up-country district hospital with a diagnosis of chronic kidney disease secondary to polycystic kidney disease for hemodialysis. He presented with a history of elevated blood pressure, abdominal swelling, and reduced urinary output for about 10 months. He suffered a hemorrhagic stroke 8 months earlier and has been using traditional medicine for his illness for over 6 months. He denied any history of familial kidney disease or terminal hematuria during his childhood.

On examination, he was alert and oriented with an elevated blood pressure (166/91 mm Hg). He had an asymmetrical abdominal distention (right > left) with multiple traditional marks over the right hypochondrium. An intraabdominal mass measuring 30 cm × 17 cm extending from the right hypochrondrium region downward to right inguinal outward to umbilicus crossing the midline was palpable. The examination of respiratory, cardiovascular, central nervous, and musculoskeletal systems was unremarkable. He had a creatinine of 1579 µmol/L (estimated glomerular filtration rate 3.9 mL/min), and his electrolyte panel revealed hyponatremia (129 mmol/L), hyperkalemia (6.7 mmol/L), and hypocalcemia (1.88 mmol/L). His full blood count was evident for a normocytic normochromic anemia (hemoglobin 6.78 g/dL). Furthermore, he had elevated triglycerides (2.46 mmol/L), low-density lipoproteins (5.22 mmol/L), and total cholesterol (6.9 mmol/L).

**Figure 1.**
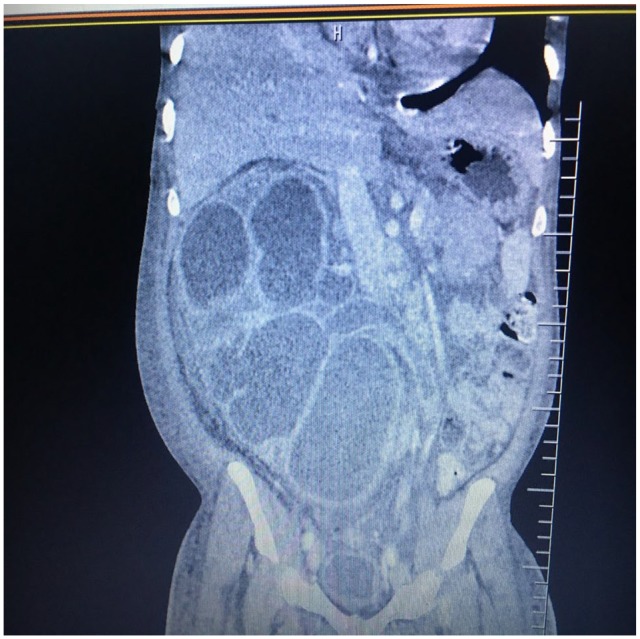
Computed tomography scan of the abdomen (coronal reformatted view) showing massive right-sided hydroureteronephrosis.

**Figure 2.**
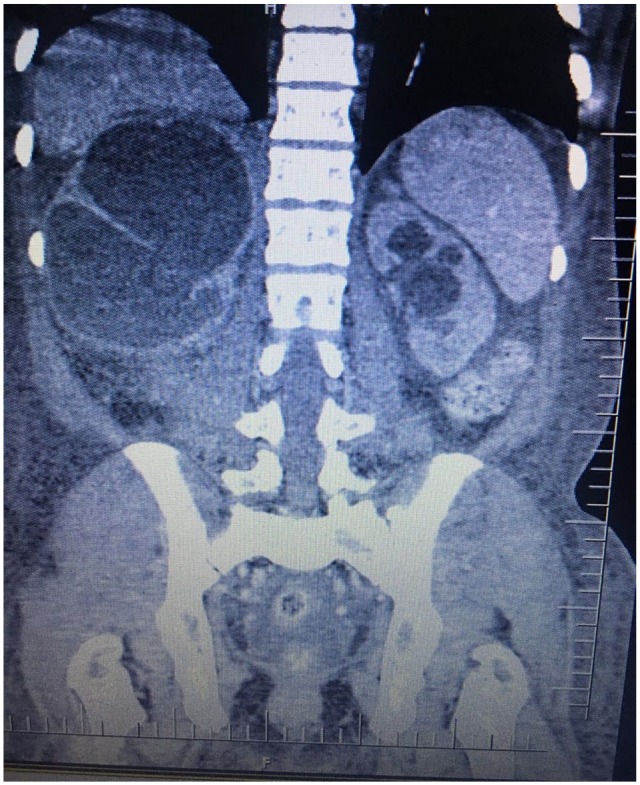
Computed tomography scan of the abdomen (coronal reformatted view) showing mild left-sided hydroureteronephrosis.

**Figure 3.**
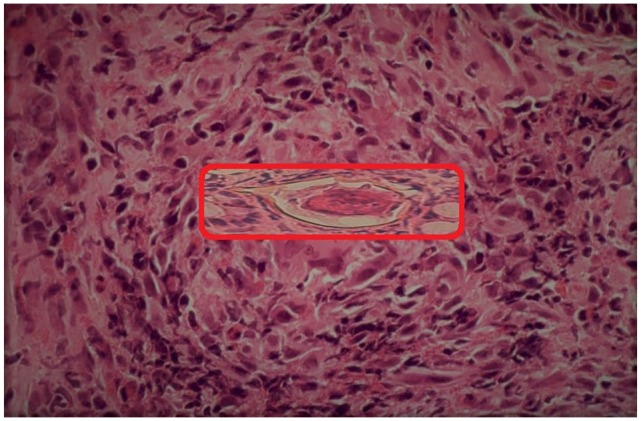
Rectal biopsy displaying *haematobium* egg.

Urinalysis revealed a urinary tract infection with a nephrotic range proteinuria. A computed tomography intravenous urogram scan of the abdomen revealed hepatomegaly (18 cm), bilateral renal enlargement with hydroureteronephrosis (right > left), and multiple calcifications on the urinary bladder (Figures 1 and 2). Microscopic examination of urine and stool samples did not detect any parasite eggs. However, a rectal biopsy isolated *haematobium* eggs and confirmed the diagnosis (Figure 3). The urinary tract infection, hypertension, electrolyte imbalances, anemia, ESRD, and schistosomiasis were managed with respective management protocols. Despite medical and symptomatic management, this patient died of renal failure on the 18th day of hospitalization.

## Discussion

Globally, schistosomal infections afflicts over 200 million people, resulting in the loss of 70 million disability-adjusted life years.^1,2,3,5,11-13^ Detection of schistosome eggs in the urine and stool is the gold standard for a definitive diagnosis. As endorsed by the World Health Organization, preventative chemotherapy through mass administration of a single oral dose of 40 mg/kg of praziquantel remains the cornerstone of schistosomiasis control.^12,13^ With the efficacy, compliance, and global coverage of ~50%, <50%, and ~20%, respectively, just about 5% of the human population benefits from praziquantel as an intermittent preventive therapy for schistosomiasis.^13^

Despite of its known devastating potential on the urogenital system, urinary schistosomiasis is often underrecognized and underevaluated. Of the 5 species known to cause disease in humans, *Schistosoma haematobium* predominantly lives in the vesical plexus near the urinary bladder making it the only specie with preferential affinity for the genitourinary system.^3^ Immunopathological reactions against schistosome eggs entrapped in host tissues may result in granulomas, fibrosis, and/or calcification of the ureter, bladder, and urethra.^4^ Ultimately, vesicoureteral reflux and obstructive consequences leading to chronic bacteriuria, hydroureter, and hydronephrosis are foreseeable.^4^ Furthermore, chronic infection with the *haematobium* species has been linked with the development of squamous cell carcinoma of the bladder.^5^ This calls for extensive evaluation of each confirmed case to prevent the progression of renal impairment to ESRD and/or death.

In conclusion, urinary schistosomiasis can have distressing effects on the urinary system in particular and survival prospects in general as demonstrated in this case report. In view of this, all cases of confirmed urinary schistosomiasis should undergo thorough hematological, biochemical, and radiological evaluation of the genitourinary system so that individuals with obstructive uropathy could be identified in a timely manner and managed promptly.
